# Supplementary material for: Mother's curse is pervasive across a large mitonuclear Drosophila panel
Source: Evol Lett. 2021 Mar 13;5(3):230–9. doi: 10.1002/evl3.221 (PMC8190446; doi:10.1002/evl3.221)
Supplement: Supplementary file 1 — Table S1: Drosophila rearing medium recipe. This recipe makes for 2L worth of food. Figure S1: Mitochondrial genetic variation for each nuclear genome and sex combination. Table S2: Mitochondrial genetic differentiation matrix. Table S3: Outputs from linear models where centroid size was modelled with mtDNA, nuDNA and sex as fixed factors. Table S4: Proportion of variance explained by explanatory variables for both male and female models. Figure S2: Centroid size correlations. Table S5: Mitochondrial genetic correlations per nuclear genome Table S6: Coefficient of variation (CV), variance and standard deviation estimates for both sexes and within each nuclear genome. Table S7: Degree of coadaptation for each nuclear genome [file EVL3-5-230-s001.pdf]

Supplementary:

Table S1: Drosophila rearing medium recipe. This recipe makes for 2L worth of food.

Ingredient	Quantity
Yeast	200g
Sugar	200g
Agar	30g
Water	2L
Propionic acid (preservative)	6mL
Nipagin (preservative)	60mL

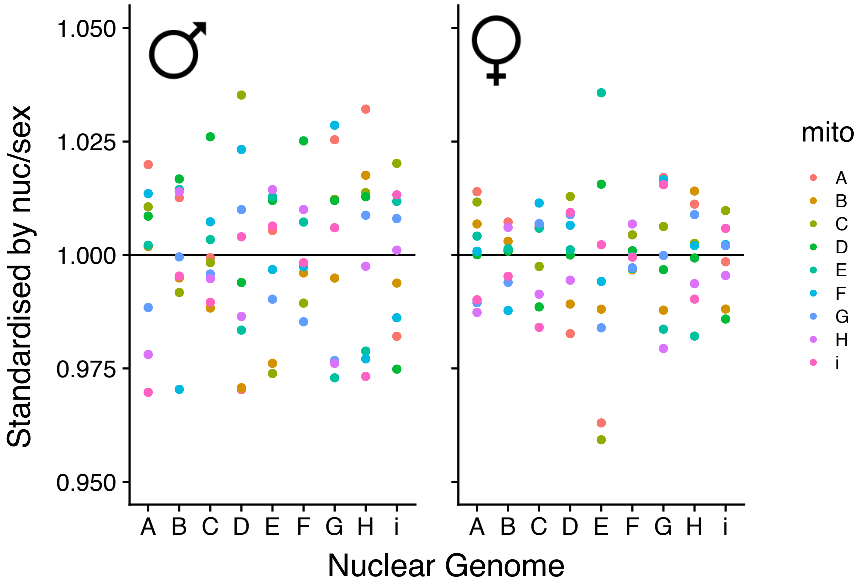

Figure S1: Mitochondrial genetic variation for each nuclear genome and sex combination. Values have been standardised by the mean nuclear genome and sex-specific mean and allows for better visualisation of the patterns observed.

Table S2: Mitochondrial genetic differentiation matrix. Below the diagonal we show the total number of SNPs difference between the mtDNA genomes, with the top of the diagonal showing the non-synonymous SNPs.

	A	B	C	D	E	F	G	H	i
**A**	-	1	0	1	1	0	0	0	4
**B**	32	-	1	2	2	1	1	1	5
**C**	27	35	-	1	1	0	0	0	4
**D**	27	35	8	-	2	1	1	1	5
**E**	25	31	22	22	-	1	1	1	5
**F**	27	37	8	12	24	-	0	0	4
**G**	29	31	20	24	14	24	-	0	4
**H**	24	26	25	25	13	27	9	-	4
**i**	62	65	56	62	58	58	56	61	-

Table S3: Outputs from linear models where centroid size was modelled with mtDNA, nuDNA and sex as fixed factors.

Full Model

	Df	Sum Sq	Mean Sq	F value	Pr(>F)
mito	8	0.17	0.0213	16.7597	< 0.001
nuc	8	3.361	0.4201	331.2966	< 0.001
sex	1	16.9016	16.9016	13328.2098	< 0.001
mito × nuc	63	0.6089	0.0097	7.621	< 0.001
mito × sex	8	0.0235	0.0029	2.3153	0.01806
nuc × sex	8	0.1194	0.0149	11.7717	< 0.001
mito × nuc × sex	63	0.1249	0.002	1.5639	0.003481
Residuals	1688	2.1406	0.0013		

Female

	Df	Sum Sq	Mean Sq	F value	Pr(>F)
mito	8	0.03279	0.004099	3.0345	0.00224
nuc	8	1.80344	0.22543	166.8931	< 0.001
mito × nuc	63	0.38261	0.006073	4.4962	< 0.001
Residuals	977	1.31968	0.001351		

Male

	Df	Sum Sq	Mean Sq	F value	Pr(>F)
mito	8	0.055	0.006875	5.9547	< 0.001
nuc	8	1.14963	0.143704	124.4664	< 0.001
mito × nuc	63	0.37472	0.005948	5.1517	< 0.001
Residuals	711	0.82089	0.001155		

Table S4: Proportion of variance explained by explanatory variables for both male and female models.

	male	female
mito	0.023 (2.3%)	0.009 (0.9%)
nuc	0.479 (47.9%)	0.510 (51%)
mito × nuc	0.156 (15.6%)	0.108 (10.8%)

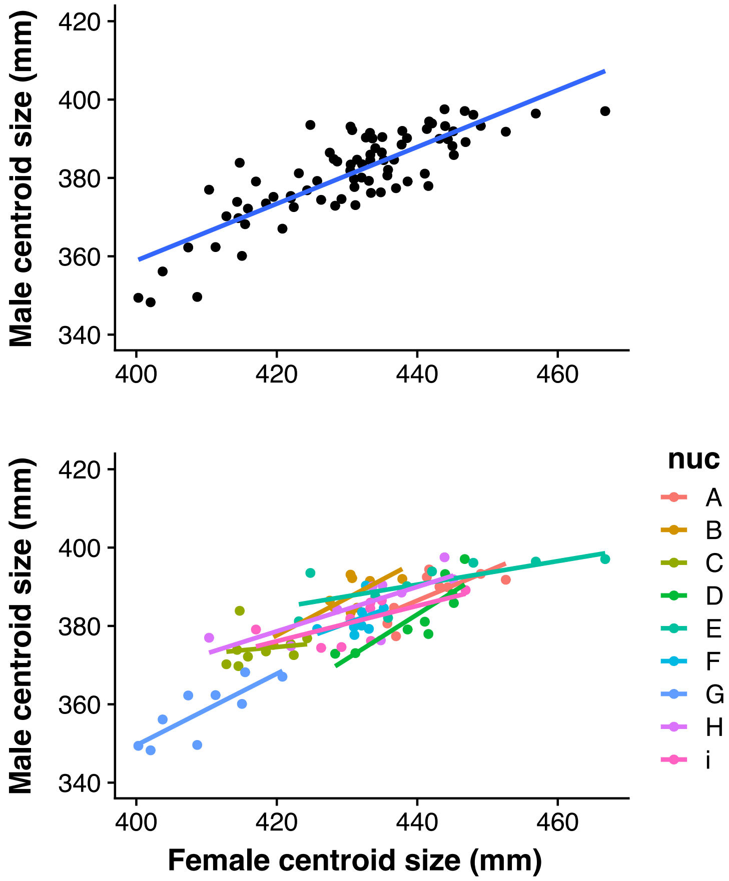

Figure S2: Centroid size correlations. We performed correlations for centroid size between males and females (top). The bottom plot shows the same correlation, but distinct nuclear genomes are coloured, for better visualization of mitochondrial genetic correlations.

Table S5: Mitochondrial genetic correlations per nuclear genome

nuc	r	p	CI	
A	0.7131035	**0.03103**	0.09304822	0.93460699
B	0.8026384	**0.009216**	0.2966411	0.9567597
C	0.1652414	0.6709	-0.5603762	0.7473488
D	0.8558237	**0.003245**	0.4441361	0.9691236
E	0.7026195	**0.03479**	0.07217775	0.93189557
F	0.4303116	0.2872	-0.393761	0.8709019
G	0.8172228	**0.007153**	0.3348195	0.9602065
H	0.7701874	**0.01517**	0.2171246	0.9489321
i	0.6448916	0.06075	-0.0336358	0.91649212

Table S6: Coefficient of variation (CV), variance and standard deviation estimates for both sexes and within each nuclear genome. Shown in bold are cases where the estimates are greater in males than females.

	CV		Variance		Standard deviation	
nuc	male	female	male	female	male	female
A	**0.01564599**	0.00905772	**0.00200078**	0.00163517	**0.04473007**	0.04043718
B	**0.01405321**	0.00576693	**0.00096923**	0.00084238	**0.03113245**	0.02902377
C	**0.01000264**	0.00904443	0.00105992	0.00134211	0.03255647	0.03663489
D	**0.02089639**	0.00933088	**0.00236049**	0.00201607	**0.04858486**	0.04490061
E	0.01441906	0.02962826	0.00148466	0.00309969	0.03853125	0.05567481
F	**0.01169987**	0.00335646	**0.00101766**	0.00096331	**0.03190078**	0.03103725
G	**0.01989798**	0.01363829	**0.00199818**	0.00138559	**0.04470103**	0.03722357
H	**0.01915078**	0.00956285	**0.00285628**	0.0014458	**0.05344417**	0.03802367
i	**0.0146611**	0.00726518	0.00103125	0.00171638	0.03211301	0.04142919

Mito-nuclear coadaptation

One of the questions we wanted answered was if coevolved genotypes had consistently larger wings than disrupted. This is under the assumption that disrupting mito-nuclear interactions would have energetic consequences and flies would be smaller. For this we calculated the differences between the average coevolved wing size and the average disrupted wing size. While we compress a lot of datapoints into a single value, it was the best strategy (to our knowledge) to avoid comparing groups with unequal sample sizes (there are 8-times more disrupted lines than coevolved).

Table S7: Degree of coadaptation for each nuclear genome

nuc	male	female
A	**0.0377**	**0.027**
B	-0.0088	**0.006**
C	-0.0029	-0.005
D	-0.0095	0.000
E	**0.0241**	**0.069**
F	-0.0045	-0.005
G	-0.0371	0.000
H	-0.0042	-0.012
i	**0.0247**	**0.013**
